# Neuronal porosome lipidome

**DOI:** 10.1111/jcmm.12383

**Published:** 2014-09-16

**Authors:** Kenneth T Lewis, Krishna R Maddipati, Douglas J Taatjes, Bhanu P Jena

**Affiliations:** a Department of Physiology, Wayne State University School of MedicineDetroit, MI, USA; b Department of Pathology, Lipidomics Core Facility, Wayne State University School of MedicineDetroit, MI, USA; c Department of Pathology, University of Vermont College of MedicineBurlington, VT, USA

**Keywords:** neuronal porosome, lipid composition, neurotransmitter release, mass spectrometry, lipid overlay

## Abstract

Cup-shaped lipoprotein structures called porosomes are the universal secretory portals at the cell plasma membrane, where secretory vesicles transiently dock and fuse to release intravesicular contents. In neurons, porosomes measure ∼15 nm and are comprised of nearly 40 proteins, among them SNAREs, ion channels, the G_αo_ G-protein and several structural proteins. Earlier studies report the interaction of specific lipids and their influence on SNAREs, ion channels and G-protein function. Our own studies demonstrate the requirement of cholesterol for the maintenance of neuronal porosome integrity, and the influence of lipids on SNARE complex assembly. In this study, to further understand the role of lipids on porosome structure-function, the lipid composition of isolated neuronal porosome was determined using mass spectrometry. Using lipid-binding assays, the affinity of porosome-associated syntaxin-1A to various lipids was determined. Our mass spectrometry results demonstrate the presence of phosphatidylinositol phosphates (PIP's) and phosphatidic acid (PA) among other lipids, and the enriched presence of ceramide (Cer), lysophosphatidylinositol phosphates (LPIP) and diacylglycerol (DAG). Lipid binding assays demonstrate the binding of neuronal porosome to cardiolipin, and confirm its association with PIP's and PA. The ability of exogenous PA to alter protein–protein interaction and neurotransmitter release is further demonstrated from the study.

## Introduction

**Figure 1:**
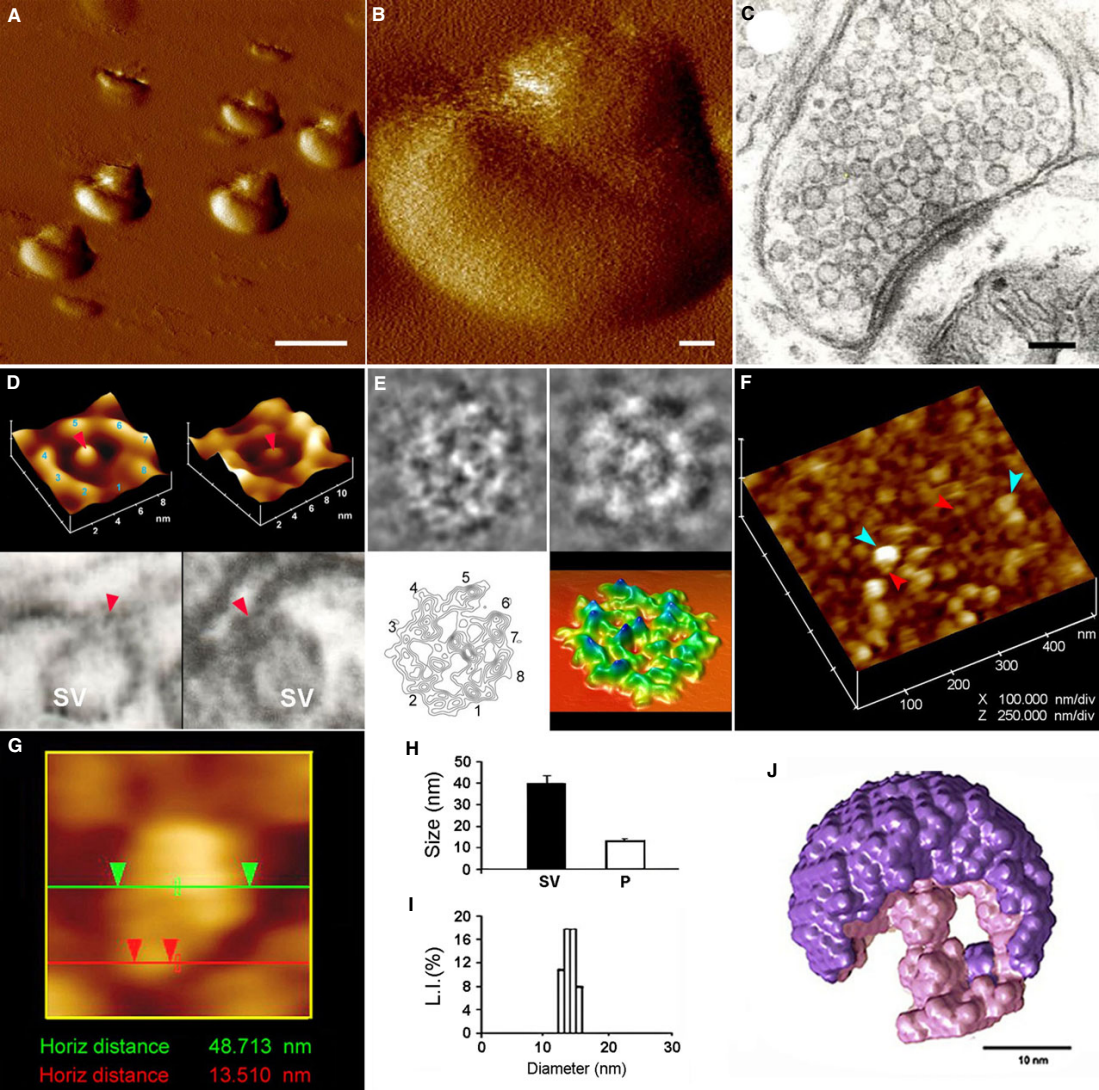
Structure and organization of the neuronal porosome complex at the nerve terminal 8,9. (**A**) Low resolution atomic force microscopy (AFM) amplitude image; bar = 1 μm (**B**) and high-resolution AFM amplitude image; bar = 100 nm (**B**) of isolated rat brain synaptosomes in buffered solution. (**C**) Electron micrograph of a synaptosome 4, bar = 100 nm. (**D**) Structure and arrangement of the neuronal porosome complex facing the outside (**D**, top left), and the arrangement of the reconstituted complex in PC:PS membrane (**D**, top right). Lower panels are two transmission electron micrographs demonstrating synaptic vesicles (SV) docked at the base of cup-shaped porosome, having a central plug (red arrowhead) 7. (**E**) Electron microscopy (EM), electron density and 3D contour mapping, provides at the nanoscale, the structure and assembly of proteins within the complex 7. (**F**) AFM micrograph of inside-out membrane preparations of isolated synaptosome. Note the porosomes (red arrowheads) to which synaptic vesicles are found docked (blue arrow head) 4. (**G**) High-resolution AFM micrograph of a synaptic vesicle docked to a porosome at the cytoplasmic compartment of the pre-synaptic membrane 4. (**H**) AFM measurements (*n* = 15) of porosomes (P, 13.05 ± 0.91) and synaptic vesicles (SV, 40.15 ± 3.14) at the cytoplasmic compartment of the pre-synaptic membrane 14. (**I**) Photon correlation spectroscopy (PCS) on immunoisolated neuronal porosome complex demonstrates their size to range from 12 to 16 nm 14. (**J**) The averaged small angle X-Ray solution scattering (SAXS) 3D structure of synaptic vesicle (purple) docked at the cup-shaped neuronal porosome complex (pink) at the pre-synaptic membrane in isolated synaptosomes, is presented 9. Note that the AFM, EM and SAXS images, all demonstrate similarity in the docking and interaction of synaptic vesicles with the neuronal porosome complex at the pre-synaptic membrane. ©Bhanu Jena.

In cells it is estimated that a staggering number of lipids, more than 10,000 different species are present 1. It is beginning to be appreciated that lipids are a major class of bio-molecules involved in almost every aspect of cellular structure-function, from cell signalling and ion channel function, to cellular and sub-cellular compartmentalization and membrane fission and fusion. Cup-shaped lipoprotein structures called porosomes 2–9 are present at the cell plasma membrane, where secretory vesicles transiently dock and fuse at its base to release intravesicular contents to the outside. Fusion of membrane-bound secretory vesicles at the porosome base is mediated by calcium and a specialized set of three soluble *N*-ethylmaleimide-sensitive factor-attachment protein receptors called SNAREs 10–13. In neurons, for example, target membrane proteins SNAP-25 and syntaxin-1A called t-SNAREs present at the base of neuronal porosomes, and a synaptic vesicle-associated membrane protein or v-SNARE, are part of the conserved protein complex involved in membrane fusion and neurotransmission. In neurons, porosomes measuring ∼15 nm are present at the pre-synaptic membrane (Fig.1), and are composed of nearly 40 proteins, among them SNAREs, ion channels such as calcium channels, and the GTP-binding G-protein G_α0_ 2–9. In Figure1, a compiled summary of some of the key structural features of the neuronal porosome complex determined from previous study 4,7–9 is presented for clarity.

Earlier studies demonstrate the requirement of cholesterol for the maintenance of neuronal porosome integrity 14. Loss of interaction between syntaxin-1A and N-type calcium channel within the porosome complex is observed following exposure to saponin 14. Similarly, the interaction of phosphatidic acid (PA) and other polyphosphoinositide (PI) lipids with syntaxin-1A, and the involvement of these lipids in cell secretion, has been reported 15. The effect of membrane lipids on ion channel function 16, including calcium channels 17, has also been reported. Studies 17 show that the activity of calcium channels could be modulated by lipid domain formation upon minor changes to the composition of membrane lipids. Recent studies involving the crystal structure of a lipid-G-protein-coupled receptor 18 demonstrates that the lyso-phospholipid sphingosine 1-phosphate modulates lymphocyte trafficking, endothelial development and integrity, heart rate, and vascular tone, by activating the G-protein-coupled receptor. These few examples clearly demonstrate the critical role of membrane lipids on various cellular functions. It is important to recognize that while minor changes in membrane lipids manifest major changes in the function of membrane proteins and protein complexes, membrane lipid composition varies between organelles and different microdomains at the plasma membrane of the same cell 16. In fact, the lipid composition differs between the inner and outer leaflet of the bilayer at a certain domain of the cell membrane 16. Therefore, to understand the role of lipids on the neuronal porosome structure-function, the determination of its lipid composition was essential. A previously published neuronal porosome isolation protocol was used that allows purification of the entire functional porosome complex 4,7–9,14. Associated lipids within the porosome complex were assessed using mass spectrometry. To further determine lipid interactions with specific porosome proteins, lipid-binding/lipid overlay assays were performed and known lipid-binding domains within the porosome complex for possible lipid-protein interactions was assessed in this study.

## Materials and methods

### Synaptosome preparation

Synaptosomes were prepared from rat brains according to published methods 4,14. For each experiment, Sprague–Dawley rats weighing 100–150 g were killed by CO_2_ inhalation, with all animal procedures preapproved by the Institution Animal Care & Use Committee (IACUC). Whole brains were isolated and placed in ice-cold buffered sucrose solution (5 mM Hepes, pH 7.4, 0.32 M sucrose) supplemented with protease inhibitor cocktail (Sigma-Aldrich, St. Louis, MO, USA). The brain tissue was homogenized using nine strokes in a Teflon-glass homogenizer, and the total homogenate was centrifuged for 3 min. at 2500 × g. The resulting supernatant fraction was further centrifuged for 15 min. at 14,500 × g, to obtain a pellet. The resultant pellet was resuspended in buffered sucrose solution, and loaded onto a 3–10–23% Percoll gradient. After centrifugation at 28,000 × g for 6 min., the enriched synaptosome fraction at the 10–23% Percoll gradient interface was collected for the study. Typically, synaptosomes were isolated at a concentration of 2.5 mg/ml, estimated using the Bradford protein assay 19.

### Immunoisolation of the neuronal porosome complex from synaptosomes

To isolate the neuronal porosome complex, SNAP-25 specific antibody (Santa Cruz Biotechnology Inc., Dallas, TX, USA) conjugated to protein A-sepharose® was utilized. Synaptosomes isolated from rat brain tissue were used for immunoisolation of the neuronal porosome complexes. For each immunoisolation of the neuronal porosome complex, 1 mg of Triton-Lubrol-solubilized synaptosome was used. Protein in the isolated synaptosome preparation was estimated by the Bradford method 19. The Triton/Lubrol solubilization buffer contained 0.5% Lubrol; 1 mM benzamidine; 5 mM Mg-ATP; 5 mM EDTA; 0.5% Triton X-100, in PBS at pH 7.5, supplemented with protease inhibitor mix (Sigma-Aldrich). Ten micrograms of SNAP-25 antibody conjugated to the protein A-sepharose® was incubated with the 1 mg of the solubilized fractions for 1 hr at room temperature followed by three washes of 10 vol of wash buffer (500 mM NaCl, 10 mM Tris, 2 mM EDTA, pH 7.5). The immunoprecipitated sample attached to the immunosepharose beads were eluted using low pH buffer (pH 3.5) to obtain the neuronal porosome complex, and the elute immediately brought to neutral pH.

### Immunoisolation of the water channel AQP1 from synaptosomes

To immunoisolate AQP1 from synaptosomes, AQP1 specific antibody (Santa Cruz Biotechnology Inc.) conjugated to protein A-sepharose® was utilized. For each immunoisolation using synaptosomes, 1 mg of Triton-Lubrol-solubilized synaptosomes was used. The Triton/Lubrol solubilization buffer contained 0.5% Lubrol; 1 mM benzamidine; 5 mM Mg-ATP; 5 mM EDTA; 0.5% Triton X-100, in PBS at pH 7.5, supplemented with protease inhibitor mix (Sigma-Aldrich). Ten micrograms of AQP1-specific antibody conjugated to the protein A-sepharose® was incubated with the 1 mg of the solubilized fractions for 1 hr at room temperature followed by three washes of 10 vol of wash buffer (500 mM NaCl, 10 mM Tris, 2 mM EDTA, pH 7.5). The immunoprecipitated sample attached to the immunosepharose beads was eluted using low pH buffer (pH 3.5) to obtain the water channel and associated lipid complex, and the eluate immediately brought to neutral pH prior to use.

### Negative staining and transmission electron microscopy

Isolated neuronal porosomes were fixed in 0.1% paraformaldehyde + 0.1% glutaraldehyde in Hepes-buffered saline (pH 7.2) and stored at 4°C. Formvar/carbon coated 400 mesh copper grids were treated with 1% alcian blue for 10 min. at room temperature, followed by four brief rinses in distilled water and air drying. Approximately 5 μl of sample solution were then deposited onto the grid surface with a pipette, followed after one minute by two rinses with 0.1 M cacodylate buffer and finally with two rinses in distilled water. The grids were then placed onto drops of 2% aqueous uranyl acetate for 1 min., followed by wicking away the staining solution from the grid with filter paper, and finally air drying. The negatively stained preparations were imaged with a JEOL 1400 transmission electron microscope (JEOL USA, Inc., Peabody, MA, USA) operating at 60 kV. Digital images were acquired with an AMT-XR611 11 megapixel CCD camera (Advanced Microscopy Techniques, Danvers, MA, USA) and saved in tiff format (12.2 mB/image).

### Estimation of major lipids in isolated synaptosomes, and immunoisolated AQP1 and neuronal porosome complex from synaptosome preparations

#### Lipid extraction for mass spectrometry

Immunoisolated neuronal porosome were extracted for lipids with methanol and methyl-tert-butyl ether (MTBE) according to published methods 20. Briefly, methanol (1.5 mL) containing 100 ng each of internal standards (diheptadecanoyl PC, diheptadecanoyl PE, diheptadecanoyl PS, diheptadecanoyl PA, diheptadecanoyl PG, diheptadecanoyl glycerol-d5, 1,3-diheptadecanoyl-2-(10Z)heptdecenoyl glycerol-d5, 1-palmitoyl(d31)-2-oleoyl-sn-glycero-3-phosphoinositol, N-heptadecanoyl C-18 ceramide, N-heptadecanoyl C-18 sphingomyelin and PAF-C16-d4) was added to a suspension of neuronal porosome (20 μl) followed by MTBE (5 ml), and mixed well. The mixture was left for 1 hr at room temperature with occasional mixing. Water (1.5 ml) was added to the mixture, mixed thoroughly, and centrifuged (1000 × g) for 5 min. to assist the separation of phases. The upper organic phase was collected to a clean glass tube. The lower aqueous phase was extracted twice (2 ml each time) with MTBE saturated with methanol and water (10:3:2.5 v/v) and the extracts were combined. The MTBE extracts were evaporated to dryness under a gentle stream of nitrogen and the residue was dissolved in LC-MS grade isopropanol-hexane-100 mM aqueous ammonium acetate (58:40:2 v/v). The reconstituted lipid extract was analysed for lipids by mass spectrometry.

#### Mass spectrometric quantitation of lipid classes

Lipid extracts were directly infused into the TurboVion source by a syringe pump at 10 μl/min. and analysed by QTRAP5500 mass spectrometer (ABSCIEX, Farmington, MA, USA). Multiple precursor ion and neutral loss scanning methods were used for Information Dependent Acquisition of ms/ms data to detect and quantify the lipid classes as described earlier. Mass analyser conditions in the positive ion mode are as follows: Ionization Potential: 5500 V, Declustering Potential: 120 V, Entrance Potential: 9 V, Collision cell Exit Potential: 9 V. Collision energy for the survey scan was 10 and 45 eV for Enhanced Product Ion scans. In each scan, three ions with highest intensity were chosen for dependent product ion acquisition and the detected ions were excluded for the rest of the experiment after three occurrences. Data were analysed for the identification of lipid species using LipidView software (ABSCIEX). Lipid were quantified against internal standards and normalized against protein values obtained by the Bradford assay 19.

### Porosome complex and lipid binding assay

Membrane lipid strips spotted with 100 pmol each of 15 different lipids are exposed to the isolated porosome, in a protein-lipid overlay assay. The bound porosome-associated protein syntaxin 1A, is identified using a specific antibody against it in a Western blot, to determine what lipid or lipids the protein has affinity for and binds to. Membrane lipid strips measuring 2 × 6 cm used in the study were obtained from Echelon Biosciences Inc. (Salt Lake City, UT, USA).

### Sucrose density gradient fractionation of solubilized control synaptosomes, and synaptosomes pre-exposed to PA

Approximately 500 μg of Triton/Lubrol-solubilized synaptosomes containing 1 mM benzamidine, 5 mM Mg-ATP, 5 mM EDTA, supplemented with protease inhibitor mix (Sigma-Aldrich), in PBS at pH 7.5 was used for each control experiment. In the experimental sets, 1 mg of isolated synaptosomes exposed to 5 μM PA for 1 hr at 4°C was used. Post PA incubation, the synaptosomes were similarly solubilized as control synaptosomes using Triton/Lubrol containing 1 mM benzamidine, 5 mM Mg-ATP, 5 mM EDTA, supplemented with protease inhibitor mix (Sigma-Aldrich), in PBS at pH 7.5. Sucrose density fractionation of solubilized synaptosomes was carried out using a minor modification of a published procedure 20. Two hundred microlitres of the solubilized synaptosome preparation was layered over a sucrose gradient prepared by successively layering 400 μl each of 50%, 40%, 30%, 20% and 10% sucrose. After centrifugation at 240,000 × g in a Beckman Coulter Optima TLX Ultracentrifuge for 3 hrs at 4°C, 200 μl fractions were collected from the top of the gradient designated fractions number 1 through 11. Equal volume of each fraction were resuspended in Laemmli 21, reducing sample preparation buffer and resolved using 10% SDS-PAGE for both Coomassie Blue staining of protein fractions and electrotransfer to nitrocellulose membrane for Western blot analysis.

### SDS-PAGE, Coomassie Blue Staining, and Western blot analysis

Protein concentration was estimated in isolated synaptosomes using the Bradford method 19. Immunoisolated neuronal porosomes and known amount of synaptosomes or solubilized synaptosome fractions in Laemmli buffer 21, were resolved in a 10% SDS-PAGE, followed by Coomassie Blue staining or processed for electrotransfer to 0.2 mm nitrocellulose sheets for Western blot analysis. The collective intensity of the stained protein bands resolved in SDS-PAGE was determined and plotted as per cent of the total optical density of all the gel bands. For Western blot analysis, the nitrocellulose was incubated for 1 hr at room temperature in blocking buffer [5% non-fat milk in PBS containing 0.1% TWEEN-20 (PBST)] and immunoblotted for 1 hr at room temperature with antibodies against syntaxin-1A and actin (Santa Cruz Biotechnology Inc.), at a dilution of 1:1000 in PBST. The immunoblotted nitrocellulose sheets were washed in PBST, prior to incubation for 1 hr at room temperature in horseradish peroxidase-conjugated secondary antibodies at a dilution of 1:5000 in blocking buffer. The immunoblots were washed in PBST and processed for enhanced chemiluminescence and exposure to RX-B film (RX-B, Denville, Metuchen, NJ, USA). The exposed films were then developed and photographed.

### Glutamate release assay from brain slices exposed to PA

Rat brain slices ∼0.5 mm thick were prepared in ice-cold PBS pH 7.4. Two brain slices were used in each assay. The brain slices were incubated for 2 hrs with or without 5 μM PA in ice-cold PBS pH 7.4. Following incubation, the brain slices were washed twice in 10 vol of ice-cold PBS pH 7.4, and finally incubated in 1.2 ml of PBS at room temperature, followed by stimulation using 30 mM KCl. Glutamate released into the medium at 0, 10, 15 and 20 min. following KCl exposure was measured using a Glutamate Assay Kit (Sigma-Aldrich Co. LLC).

## Results and discussion

**Figure 2:**
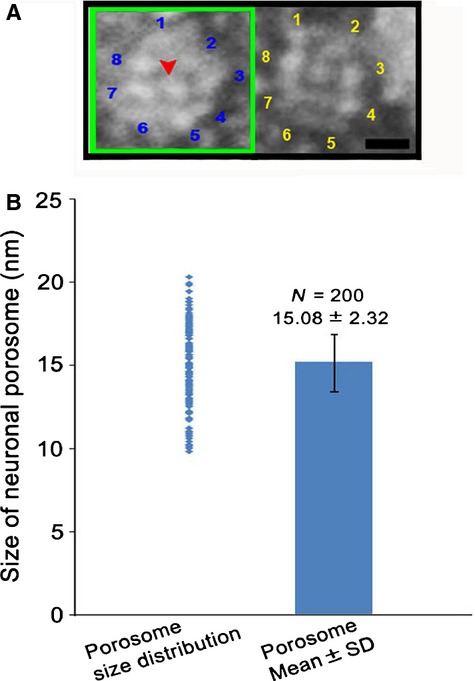
Transmission electron micrograph of negatively stained neuronal porosome complexes associated with limited lipid. (**A**) Electron micrograph of two representative images of immunoisolated neuronal porosomes demonstrating the presence of their eightfold symmetry and the central plug (bar = 5 nm). (**B**) Measurement of the diameter of isolated porosomes in electron micrograph demonstrates their size to range from 9 to 22 nm, with a mean size of ∼15 nm. Only intact and freestanding porosomes were randomly picked for measurement from several electron micrographs. Broken and clustered porosomes, the clustering most likely resulting from paraformaldehyde cross-linking because of fixation, were excluded from our reported measurements.

**Figure 3:**
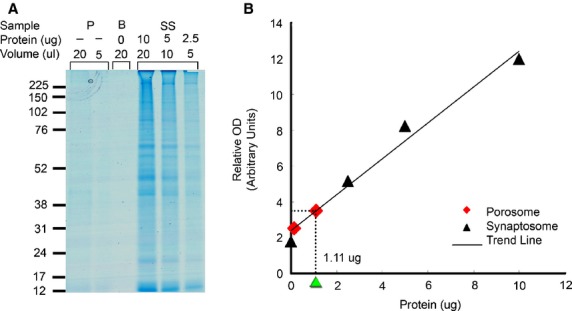
Estimation of the protein concentration, in immunoisolated neuronal porosome preparations. (**A**) Coomassie Blue stained proteins in 20 and 5 μl porosome (P) preparations, 20 μl of blank (B) containing just sample preparation buffer, and 10, 5 and 2.5 μg of isolated synaptosomes (SS), resolved using SDS-PAGE. (**B**) Plot of the known protein concentrations determined by Bradford assay (19) in SS fractions and their respective optical density in coomassie stained SDS-PAGE compared. Note the optical density of the 20 μl porosome fraction lane translates to 1.11 μg of protein.

**Figure 4:**
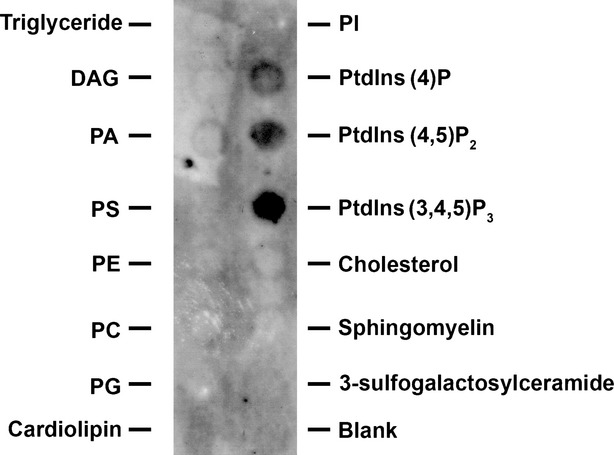
Binding of isolated neuronal porosome to specific lipids. Membrane lipid strips spotted with 100 pmol each of 15 different lipids when exposed to porosome proteins and immunoblotted using syntaxin-1A specific antibody, demonstrate bind of the porosome to PA, Cardiolipin, PIP, PIP2 and PIP3, in increasing order.

Neurotransmission is dependent on the transient fusion of 40–50 nm in diameter membrane-bound synaptic vesicles containing neurotransmitters at the base of 15 nm neuronal porosomes present at the pre-synaptic membrane in nerve terminals. Negatively stained electron micrographs of native lipid-associated neuronal porosome preparation from rat brain synaptosomes, demonstrates the immunoisolation of the complex with its known eightfold symmetry and a central plug (Fig.2) 4,7–9,14 that regulates pore dynamics 9. To determine the protein concentration of isolated porosome complex used in the lipidomic study, synaptosome preparations of known protein concentrations, and the isolated porosomes of unknown protein concentrations were resolved using SDS-PAGE and stained using Coomassie Blue (Fig.3A). A plot of the known protein concentration in the synaptosome preparation and its respective optical density provided the protein concentration (0.056 μg/μl) in the immunoisolated porosome preparation (Fig.3B). As opposed to a protein concentration of 2.4 μg/μl of the synaptosome preparation, the porosome preparation was determined to be 43-fold less (Fig.3B). Hence, the protein concentration of synaptosomes used in the lipidomic analysis was 43-fold more than the neuronal porosome used in the study. Mass spectrometry of the immunoisolated neuronal porosome complex, demonstrates the presence of phosphatidylinositol phosphates (PIP's) and PA among other lipids, and the enriched presence of ceramide (Cer), lysophosphatidylinositol phosphates (LPIP) and diacylglycerol (DAG) (Table1). The specific association of PI, PIP and PIP2 in the neuronal porosome complex, and their absence (undetectable) in the AQP1 immunoisolates is demonstrated (Table1). Lipid binding assays demonstrate the binding of neuronal porosome to cardiolipin, and confirm its association with PIP's and PA (Fig.4).

**Table 1:** Major lipid species associated with the neuronal porosome complex identified using mass spectrometry

Lipid species	Porosome	Synaptosome	AQP1
Cer (ceramides)	**4.82**	0.00	0.00
Cer-P (ceramide phosphates)	**0.48**	0.00	0.00
LPC (lysophosphatidylcholines)	0.00	0.20	0.00
PC (phosphatidylcholines)	4.94	18.50	13.62
SM (sphingomyelins)	0.00	13.78	0.07
LPG (lysophosphatidylglycerols)	0.00	0.50	0.00
LPIP (lysophosphatidylinositol phosphates)	**23.61**	2.81	15.45
LPIP2 (lysophosphatidylinositol diphosphates)	**0.34**	0.14	0.00
LPS (lysophosphatidylserines)	0.00	0.32	0.00
PA (phosphatidic acids)	0.40	2.53	0.00
PE (phosphatidylethanolamines)	0.00	0.94	0.00
PG (phosphatidylglycerols)	0.34	5.19	0.00
PI (phosphatidylinositols)	0.74	6.86	0.00
PIP (phosphatidylinositol phosphates)	1.61	3.23	0.00
PIP2 (phosphatidylinositol diphosphates)	2.98	5.16	0.00
PS (phosphatidylserines)	2.31	20.36	2.21
DAG (diacylglycerols)	**393.74**	2.12	675.89
MADAG (1-alkyl-2-acylglycerols)	**3.00**	0.51	0.00
TAG (triacylglycerols)	0.00	0.19	0.00

Both the porosome and AQP1 (control) were purified from isolated synaptosome preparation. Note the enriched presence of Cer, Cer-P, LPIP, LPIP2, DAG and MADAG in the isolated neuronal porosome complex. Also note the specific association of PI, PIP and PIP2 in the neuronal porosome complex, and their absence in the AQP1 immunoisolates. Protein normalized lipid data are presented as ng/μg protein.

Some of the key proteins composing the neuronal porosome complex identified earlier 8 using a combination of mass spectrometry and Western blot analysis are: actin, tubulin, spectrin, dystrophin, langerin, GTPase activating protein GAP, intersectin, myosin, Na^+^/K^+^-transporting ATPase, Ca^+^-transporting ATPase, G_α0_, vesicle fusing ATPase, rab3A, SNAP25, syntaxin 1A, syntaxin 1B, synapsin-2 and synaptotagmin-1. As previously outlined, earlier studies 14 demonstrate the requirement of cholesterol for maintaining the integrity of the neuronal porosome. It is demonstrated that loss of interaction between syntaxin 1A and the N-type calcium channel within the neuronal porosome complex is observed following exposure to β-cyclodextran 14. Similarly, the interaction of PA and other PI lipids with syntaxin-1A, and the involvement of these lipids in cell secretion, has been reported 15. In the Lam *et al*., study 15, it was demonstrated that PA a fusogenic lipid, directly binds to a polybasic juxtamembrane region within syntaxin-1A. Syntaxin-1A mutations, that progressively reduce lipid binding, were found to result in a progressive loss in cell secretion 15. In addition, overexpression of the PA-generating enzyme phospholipase D1, rescued the secretory defects in the mutants 15. Syntaxin-1A being one of the neuronal porosome proteins 8, the enriched presence of PA (Table1 and Fig.4) was informative and a further confirmation of the earlier findings 15. Similarly, studies report that syntaxin-1A clustering is also mediated by electrostatic interactions with the strong anionic lipid phosphatidylinositol-4, 5-bisphosphate (PIP2) 22. In this study by Bogaart *et al*., 22, it is further reported that in such syntaxin-1A microdomains, the PIP2 is primarily present at the inner leaflet of the membrane 22. In agreement, we find the enriched presence of PIP2 in the neuronal porosome complex (Table1), and the specific association of the complex with PIP2 (Fig.4). The binding of isolated neuronal porosome to PA, Cardiolipin, PIP, PIP2 and PIP3, in increasing order in the lipid overlay assay as observed in Figure4, is by no means a quantitative measurement. Since mass spectrometry (Table1) demonstrate the pre-association of PA, PIP and PIP2 with the porosome complex, it is likely that the pre-association of the lipids with proteins in the porosome complex could affect their binding to the lipids on the membrane.

In recent years, at least 11 lipid-binding domains have been identified in proteins 23, which include C1 24, C2 25, PH 26, FYVE 27, PX 27, ENTH 28, ANTH 28, BAR 28, FERM, PDZ and tubby domains 23 (Table2). Some possible lipid-protein interactions are also conservatively depicted in Table2. The lipid-binding C2 domain is present in the porosome-associated protein synaptotagmin. The 3D structure of the C2 domain of synaptotagmin has been reported 29. The domain forms an eight-stranded beta sandwich constructed around a conserved 4-stranded motif, designated a C2 key 29, and calcium binds to the cup-shaped depression formed by the N-and C-terminal loops of the C2-key motif. Structural analyses of several C2 domains have shown them to consist of similar ternary structures in which three Ca^2+^-binding loops are located at the end of an 8-stranded anti-parallel beta sandwich. Another porosome protein 8 spectrin beta, represent an important group of actin, dystrophin and lipid binding proteins involved in membrane anchoring 30. Similarly dystrophin, the cytoskeletal porosome protein 8 that confers resistance to contraction-relaxation cycles by interacting with other cytoskeletal and membrane proteins, possess 24 spectrin-like repeats and phospholipid binding domains 31. The effect of membrane lipids on ion channel function 16, including calcium channels 17, has also been reported. Studies 17 show that the activity of calcium channels could be modulated by lipid domain formation upon minor changes in the composition of membrane lipids. Recent studies involving the crystal structure of a lipid-G-protein-coupled receptor 18 demonstrate that the lyso-phospholipid sphingosine 1-phosphate modulates lymphocyte trafficking, endothelial development and integrity, heart rate and vascular tone by activating the G-protein-coupled receptor 18. In this way, the structure-function of several neuronal porosome proteins could either be directly or indirectly modulated by a variety of membrane lipids.

**Table 2:** List of neuronal porosome proteins containing various lipid-binding domains

Lipid-binding domains	Known proteins containing specific lipid binding domains	Lipids binding specific protein domains	Porosome proteins with lipid-binding domains
C1 (Cystein-rich C1 domains)	PKC isoforms -Diacylglycerol Kinase -c-Raf Ser/Thr Kinase -n-Chimaerin Rac-GTPase Activating Protein	Diacylglycerol or phorbol esters	
C2 (Calcium-dependant lipid-binding domain)	-cPLA2 -Phosphatidylinositol 3- kinase (PI3K) -Tensin phosphatase -Synaptotagmin 1	Wide range of lipid selectivity including PC, PS	Synaptotagmin 1
PH (Pleckstrin Homology Domain)	Phospholipase C-δ, mSos1, RasGAP, Tsk, pleckstrin, Btk, Grp1, Akt, DAPP1, TAPP1, Spectrin beta, Dystrophin	PI-(4,5)-P2; PI-(3,4,5)-P3 PI-(3,5)-P2; Ins(1,4,5)P3	GAP, Spectrin beta, Dystrophin (central rod made of 24 spectrin-like repeats)
FYVE (Fab-1, YGL023, Vps27 and EEA1 domain is a cysteine-rich Zn^2+^-binding domain of 60–70 AA)	-EEA1 Early Endosome Antigen -Hrs Putative ATPase -SARA (Smad Anchor for Receptor Activation) -FENS1	PI(3)P	
PX (Phox homology domain is ∼120 AA)	Found in more than 250 proteins, including the p40^phox^ and p47^phox^ components of the NADPH oxidase complex, sorting nexins, phospholipases D1 and 2 and the kinases PI3K and CISK.	PI(3)P; PI-(3,4)-P2 PI-(3,4,5)-P3; PI-(3,5)-P2 PI(5)P	
ENTH (Epsin N-terminal homology domain)	-CLINT1 (clathrin interactor)	PI-(4,5)-P2 PI(1,4,5)P3	
ANTH (AP180 N-terminal homology domain)		PI-(4,5)-P2	
BAR (Bin/Amphiphysin/Rvs)	Endophilins, Dynamin, Nexins, Amphiphysin, RhoGAPs, RhoGEF	Bind lipid membrane with high curvature	GAP, Dynamin Intersectin 1 (Rho family GEF)
FERM (Named after four proteins from which it was originally described: Band 4.1 (F), Ezrin >(E), Radixin (R) and Moesin (M))	Talin, Radixin, ERM, Willin, Moesin, PTP1, Radixin/Ezrin, JAK123	IP3, PI(3)P; PI(4)P; PI(5)P; PI-(4,5)P2; PI-(1,4,5)P3	
Tubby domains	Binds basic protein pockets	PIP2	

All membrane proteins are known to be solvated by a shell of lipid molecules that interact with the membrane-penetrating surface of the protein, referred to as annular lipids 32. Similarly, lipid molecules that are also found bound between transmembrane α-helices of membrane proteins within the bilayer, are referred to as non-annular lipids 32. A number of studies 33–35 investigating the crystal structures of membrane proteins report the presence of non-annular bound lipids and cholesterol molecules between transmembrane domains of membrane proteins. Annular lipid binding is dependent on the fatty acyl chain lengths, suggesting that hydrophobic matching between membrane proteins and the surrounding lipid bilayer exerts physical distortions on the α-helical bundle of membrane proteins 32. It has also been demonstrated that annular lipids readily exchange with the bulk bilayer lipids; however, the mobility of non-annular lipids is somewhat restricted 36. Hence, the precise interaction between proteins and lipids profoundly influence each other's structure-function. For example, studies report that Na-K-ATPase also present in the neuronal porosome complex 8 is highly dependent on acid phospholipids, especially phosphatidylserine (PS) for its stability 37–39. Recently, the PS-binding site of Na-K-ATPase has been identified to be located between the 8, 9 and 10 transmembrane segments of the α-subunit of the protein 40,41. In addition, recent studies 36 demonstrate the binding and activation of Na-K-ATPase by neutral phospholipids such as phosphatidylcholine (PC) or phosphatidylethanolamine (PE). Similar to Na-K-ATPase, another neuronal porosome protein Ca-ATPase 8 has recently been demonstrated to possess specific sites that bind PE 42. The absence of PE in our lipidomic analysis (Table1) may reflect the presence of PE in low abundance compared to the reported species in isolated neuronal porosomes, and hence undetectable by mass spectrometry. Significant contribution to cellular and sub-cellular structure is known to be imparted by those proteins that cross-link actin, a neuronal porosome protein 8 filaments, or connect actin filaments to cellular membranes. The spectrin superfamily of cytoskeletal proteins including spectrin β and dystrophin, known neuronal porosome proteins 8, possess Pleckstrin homology (PH) domains that bind lipids, and help serve as membrane anchors 43–46. PH repeats are each composed of up to nearly 120 amino acids, and to their basic residues, phosphates of phosphoinositides such as PI-(4,5)-P2, PI-(3,4,5)-P3, PI-(3,5)-P2 and Ins(1,4,5)P3, bind (Table2) 47. Our observation of the presence of PIP2 in the porosome complex (Table1) and in our lipid binding experiments (Fig.4) supports such protein-lipid interactions within the neuronal porosome complex, and supports the interaction of PA and other PI lipids with syntaxin-1A, and the involvement of these lipids in cell secretion as previously reported 15. Similarly, the porosome-associated proteins GAP, Dynamin and Intersectin 1 8 possess BAR (Bin/Amphiphysin/Rvs) domains, which are known to bind lipid membranes having high curvature 48. A typical BAR dimer is composed of a banana-shaped group of six helices having positive charges at the tips and along its concave surface that may mediate phospholipid binding. While BAR domains bind curved phospholipid membrane segments, it is proposed that a subset of BAR domains could physically assist in inducing membrane curvature.

**Figure 5:**
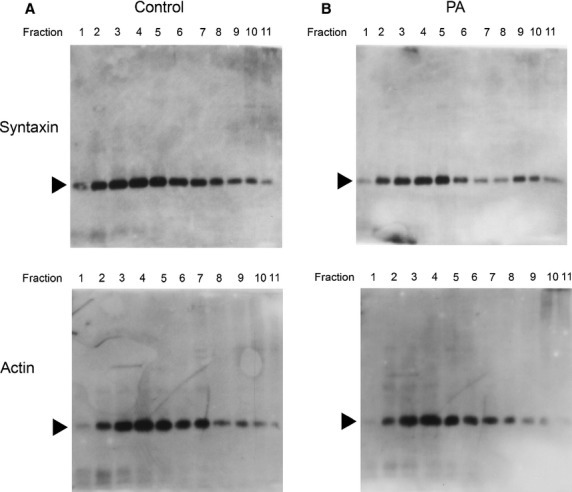
Exposure of isolated synaptosomes to phosphatidic acid (PA) alters synaptosomal protein–protein interactions. Solubilized synaptosomes with and without pre-exposure to PA followed by sucrose density gradient separation, followed by Western blot analysis, demonstrate altered distribution of the porosome-associated protein syntaxin-1A. In contrast, little change in actin distribution (another porosome-associated protein) is observed.

**Figure 6:**
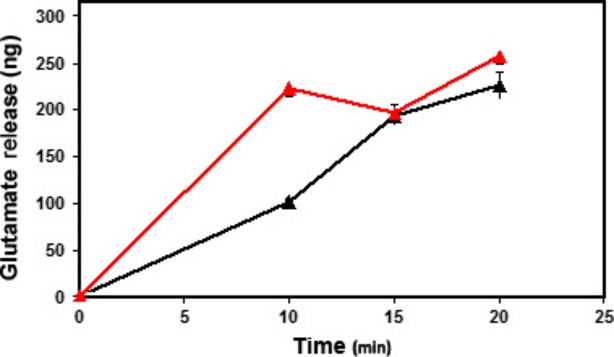
Exposure of brain slices to phosphatidic acid (PA) increases both the potency and efficacy of KCl-stimulable glutamate release. Equal amounts of brain tissue were used in all assays. Note at the 10 min. time-point, the fold increase in the potency and efficacy of glutamate release from PA-exposed brain tissue (RED) over control (BLACK). Data represent mean ± SEM;*n* = 4.

To test whether lipids influence protein–protein interaction in synaptosomes, one of the porosome-associated lipids PA was used. Synaptosomes exposed to 5 μM PA resulted in a alteration in protein distribution in synaptosomes, specifically altering the distribution of one of the two porosome-associated proteins namely syntaxin-1A (Fig.5). While little to no change in the distribution of actin immunoreactivity is seen, clearly there is a shift of syntaxin 1A immunoreactivity into the lighter membrane fraction at the top of the gradient (Fig.5). The lighter fraction would compose membrane fractions including the synaptosomal membrane, and PA may be inducing tight association of syntaxin 1A with the porosome complex. Next to test the effect of PA on synaptosome function, the release of the neurotransmitter glutamate was assayed. The release of glutamate following PA exposure was studied, since glutamate is the predominant excitatory neurotransmitter in the mammalian brain. To determine if PA exposure results in altered neuronal function, rat brain slices were subjected to a 2 hrs exposure to 5 μM PA, followed by assessment of their ability to release glutamate upon stimulation by 30 mM KCl. Results from the study demonstrate a near threefold increase in the potency and efficacy of glutamate release at the early time-point (10 min.; Fig.6), which plateau at later time-points, possibly because of high-affinity glutamate transporter function, reducing extracellular glutamate which is toxic at high concentrations, and or because of a concomitant increase in inhibitory synaptic function. In this brain slice study; however, both neurons and glia would account for the observed glutamate release following KCl stimulation. It is clearly apparent from these studies that in addition to other lipids like Cer, LPIP and DAG (Table1), the role of PA and inositol lipids on the structure-function of the neuronal porosome complex cannot be over emphasized. This is not surprising, since the majority of the lipid-binding domains in membrane proteins identified so far, bind to inositol lipids 49 (Table2), and their number is rapidly on the rise. It is of interest to note that most of the inositol lipid-binding protein domains also bind specific proteins hence protein-lipid-protein interactions could influence one another. A careful comparison of the structural features of lipid-binding domains of membrane proteins, demonstrate a high degree of conservation, reflecting on their evolutionary link and interactions 50. The important observation and advances made in the past 40 years regarding PI-(3,4,5)P3 as cellular second messengers 49 has further revealed their involvement in diverse cellular processes, including perhaps the structure and function of the neuronal porosome complex.

Ongoing experimental approaches to determine protein–protein and protein–lipid interactions involve chemical cross-linking and mass spectrometry. Experimental studies also involve high-resolution atomic force microscopy (AFM), electron microscopy (EM) and small angle X-Ray solution scattering (SAXS). Computational approaches are being employed in homology modelled protein–protein interactions and fitting studies, as well as coarse-grained docking and MD simulations to determine curvature-driven lipid sorting at the porosome complex. Results from these studies in addition to shedding light on the overall architecture of the neuronal porosome complex, the role of key porosome proteins in its function, and the role of specific porosome lipids involved in the establishment of the fusion pore, will help in progressing our understanding of the molecular processes involved in porosome-mediated neurotransmitter release at the nerve terminal.
